# Retraction and Replacement of: Aftersensations and Lingering Pain After Examination in Patients with Fibromyalgia Syndrome

**DOI:** 10.1093/pm/pnaf079

**Published:** 2025-08-06

This is a retraction and replacement of Richard J. Berwick, David A. Andersson, Andreas Goebel, Andrew Marshall, Aftersensations and Lingering Pain After Examination in Patients with Fibromyalgia Syndrome, *Pain Medicine*, Volume 23, Issue 12, December 2022, Pages 1928–1938, https://doi.org/10.1093/pm/pnac089.

In December 2024, the authors notified the journal that during a recent review of the data and figures presented in this manuscript, as part of an assessment of a larger cohort, they identified an error that necessitated a correction. A database inaccuracy affected a small subset of individuals in the PainDETECT score calculation.

The following revisions have been made:

1. Figure 2 has been replaced to reflect the corrected data.
2. Table 2 has been updated with the corrected data.
3. Supplementary Tables have been updated with the corrected data.
4. The text in the article that describes Figure 2, and sections in the methods and discussion, have been minimally updated to reflect the corrected data.

These updates alter a positive correlation to no correlation, with respect to PainDETECT score and Brushstroke Pleasantness. The updates do not affect the overall narrative, abstract, or conclusions of the manuscript.

The Authors are retracting the original version of the article and replacing it with a new version, which has been accepted by the editors. The new version has been republished as https://doi.org/10.1093/pm/pnaf080. The Authors apologize for any inconvenience caused.

This retraction and replacement is to correct the scholarly record and in no way suggests any misconduct on the part of the authors.

